# Purification of Sucrose in Sugar Beet Molasses by Utilizing Ceramic Nanofiltration and Ultrafiltration Membranes

**DOI:** 10.3390/membranes10010005

**Published:** 2019-12-27

**Authors:** Mikael Sjölin, Johan Thuvander, Ola Wallberg, Frank Lipnizki

**Affiliations:** 1 Department of Chemical Engineering, Faculty of Engineering, Lund University, 221 00 Lund, Sweden; mikael.sjolin@chemeng.lth.se (M.S.); Ola.Wallberg@chemeng.lth.se (O.W.); 2 Department of Food Technology, Engineering and Nutrition, Faculty of Engineering, Lund University, 221 00 Lund, Sweden; johan.thuvander@food.lth.se

**Keywords:** molasses, sugar beet, sucrose, ceramic membrane, ultrafiltration, nanofiltration, purification, membrane fouling and cleaning

## Abstract

Molasses is a sugar mill by-product with low value that today is used primarily for animal feed. However, molasses contains large amounts of sucrose which, if purified, could be used for other purposes. In this study, purification by membrane filtration using ceramic tubular ultrafiltration (UF) and nanofiltration (NF) was examined. NF purifies sucrose by removing small compounds, whereas UF removes larger compounds. Based on our results, high filtration fluxes could be obtained, and it was possible to clean the membranes sufficiently from fouling compounds. Sucrose was separated from other compounds, but the separation efficiency was generally higher with diluted molasses compared with concentrated molasses. This could be explained by more severe fouling when filtering dilute molasses or potentially due to aggregate formations in the molasses as our analysis showed. Overall, this study shows the potential of ceramic UF and NF membranes for sucrose purification from molasses.

## 1. Introduction

Sugar beet molasses is a low-value by-product produced from sugar mills and primarily used as animal feed and partly for bulk biofuel/biochemical fermentation [1,2]. A large part of the molasses consists of the main product of the sugar mill, sucrose, and it would be desirable if this could be used for higher-value purposes other than animal feed [3]. The sucrose could potentially be either recirculated back to the main stream of the sugar mill to be reprocessed or used in other processes such as biotechnological processes producing high value-added products [4,5]. However, molasses contains contaminants, such as polyphenols and inorganic salts, that can inhibit the growth of certain microorganisms, which requires the purification of the molasses before use in the processes [6,7].

Membrane processes have previously been demonstrated to be reliable separation processes in the food industry, based on their many advantages, such as their compact design, the possibility to handle heat-sensitive materials, and their high-energy efficiency. Ultrafiltration (UF, see Table A1 in the Appendix A for a complete list of all abbreviations) can remove high-molecular-mass contaminants from molasses, such as polyphenols, proteins, and other macromolecules, whereas nanofiltration (NF) can be used for the removal of small-molecular-mass compounds, such as salts. The major drawback of membrane processes in this field is the severity of fouling due to the presence of starch, pectin, and proteins. This highlights the importance of examining the fouling and cleaning of membranes when performing filtration for these types of raw materials [8].

Due to their temperature-stable properties, ceramic membranes are of interest investigating with regard to this separation. Furthermore, ceramic membranes can achieve high filtration fluxes and have a high chemical and mechanical resistance, which lead to longer membrane life time and process robustness in comparison to polymeric membranes. The main disadvantage of ceramic membranes is that they are generally more expensive than polymeric membranes [9]. Previously, it has not been possible to purify and concentrate sucrose and other very-small-molecular-weight compounds simultaneously using ceramic membranes, but based on recent advances in the development and commercialization of such low-molecular-weight cutoff (MWCO) ceramic NF membranes, this could now be feasible. Thus, it is of high interest to investigate how these new ceramic NF membranes can be used for various processes and feedstock solutions, and at the same time to compare their properties with a more conventional ceramic UF membrane, which removes large compounds efficiently.

In this study, an NF membrane with 200 Da MWCO and a UF membrane with 10 kDa MWCO were examined with regard to their purification of two concentrations of molasses respectively. In a larger context, the sucrose in the molasses is to be used for 5-hydroxymethylfurfural (5-HMF) production by an enzymatic bio-processes combined with a dehydration process. 5-HMF is an intermediate product which has great potential for creating building blocks for polymerization processes. The goal was to purify sucrose from potential process inhibiting compounds to an acceptable level for this downstream bio-process [10,11].

Other membrane filtration studies of similar sugar solutions have previously been performed. However, none of the previous studies have tried to use a ceramic NF membrane for retaining sucrose, which could be beneficial with regard to filtration flux and the thermo-chemical stability. A similar membrane filtration study was performed recently by Guo et al., but using cane molasses instead of sugar beet molasses and polymeric membranes instead of ceramics. Their main purpose was also to undertake a decoloration, which was not in the scope of our study [12]. There are more studies focusing on cane molasses decoloration and clarification. For example, Yang et al. tried to find a threshold flux for clarification of cane molasses in a concentration study, using polymeric UF [13]. Qiang et al. used UF as a prefiltration step, followed by a resin adsorption step and then a polymeric NF membrane, for a biorefinery utilization concept of cane molasses [14]. Luo et al. also focused on the cane molasses, but combined concentration steps with diafiltration in a successful way, using polymeric NF membranes. However, they also pretreated the feed in several steps (centrifugation, pH-adjustments, UF decoloration, etc.) [15]. Bernal et al. and Djordjević et al. studied beet molasses, but with the objective of decoloration using microfiltration/UF membranes, in combination with other process technologies [16,17]. Membrane filtration of cane molasses has also been investigated as a pretreatment step upstream fermentation processes [18,19]. Otherwise, different model solutions to separate sucrose from monosaccharides have been widely studied [20,21,22,23]. The membranes used for filtration of model solutions appear to work well for the purification of sucrose. Nevertheless, molasses is in reality a much more complex mixture of compounds, many of which may interfere with the filtration and membrane surface, or compounds that could interact with each other in different ways, as the results in this article will show.

The results of previous studies are summarized in Table 1, which highlights that most studies have been performed on cane molasses or model solutions, and when the objective was to retain sucrose using NF, mainly polymeric membranes has been used. In our study, we want to focus on beet molasses and investigate ceramic membrane performance for this application.

This study had three main objectives:

1. Determine the process parameter settings for achieving high filtration fluxes for the UF and NF of sugar beet molasses.
2. Examine the retention of various compounds of interest, depending on the process parameter settings, for both the UF and the NF.
3. Study the effects of fouling and cleaning of the membranes.

## 2. Materials and Methods 

### 2.1. Membranes and Filtration Test Scheme

The membrane in the NF trial was a ceramic tubular Fraunhofer LC1 (Fraunhofer IKTS, Hermsdorf, Germany), made of TiO_2_ as an active layer on an α-Al_2_O_3_ support with a specified MWCO of 200 Da. The tubular membrane was a lab-scale membrane (250 mm long, 10 mm d_outer_, 7 mm d_inner_) with a filtering surface of 48.4 cm^2^. For the UF experiments, an Atech tubular ceramic UF membrane was used (UF Type 37/3.8). The membrane had a MWCO of 10 kDa and consisted of TiO_2_ as the active layer on α-Al_2_O_3_ as support material. The experiments were conducted on a small pilot scale (1.2 m long tube with 37 channels, each with an inner diameter of 3.8 mm) with a total filtration area of 0.53 m^2^.

**Figure 1:**
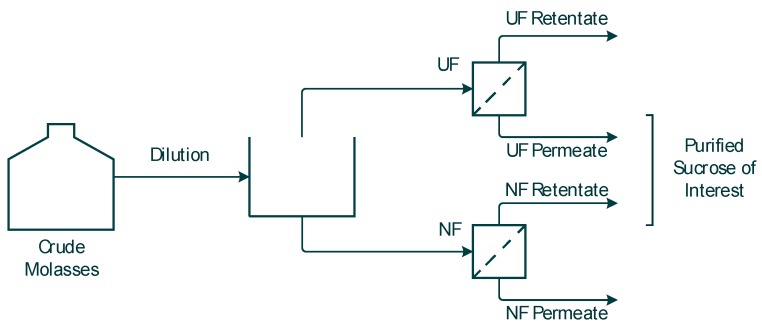
Schematic picture of the experiments performed.

Both the NF and the UF membrane performances were investigated with the goal to obtain purified sucrose for future 5-HMF synthesis. The feedstock samples were collected from the UF permeate and NF retentate, as schematically illustrated in Figure 1. It is expected that the UF membrane will retain larger molecular complexes, while the NF membrane will retain the sucrose while smaller compounds, such as salts and organic acids, will be separated out in the permeate.

### 2.2. Experimental Setup of Nanofiltration Trials

**Figure 2:**
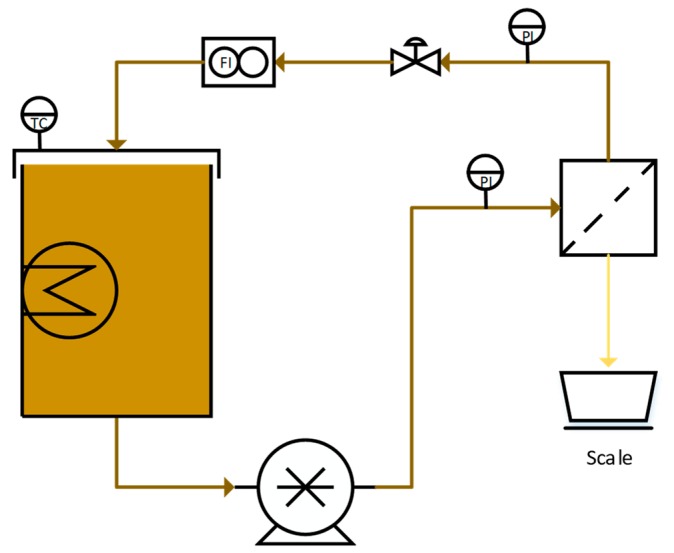
Experimental setup of the nanofiltration (NF) trials.

The NF parameter study was performed in crossflow batch mode with recirculating retentate flow, using the equipment schematically shown in Figure 2. A 15 L tank was filled with the feed solution and equipped with an immersion heater (Backer, Elektro-Värme, Sösdala, Sweden), which was regulated thermostatically by a control unit (Model MCM, Shinko Technos Co., Ltd., Osaka, Japan). The flow was then set with a positive displacement pump (Hydra-cell D25XL, Wanner, Minneapolis, MN, USA) that was controlled by a frequency converter (ELEX 4000, Bergkvist and Co. AB, Gothenburg, Sweden). The pressure was measured by two digital pressure gauges (DCS40.0AR, Trafag AG, Bubikon, Switzerland) on the feed and retentate sides, respectively, and was adjusted with a needle valve on the retentate side. The flow was measured using a flowmeter (FCH-34-PP-Chemical, B.I.O-TECH e.K., Vilshofen, Germany), and the flux was determined by measuring the weight of the permeate flow on a scale (PL6001-S, Mettler Toledo Inc., Columbus, OH, USA). The temperature, flux, pressures, and flow were monitored and logged using Labview 2009 software (National Instruments Co., Austin, TX, USA).

Two concentrations of molasses were tested, one low-concentration molasses (LCM) trial and one high-concentration molasses (HCM) trial. The LCM and HCM were prepared by diluting molasses to 200 and 10 times its original weight using deionized water, resulting in 0.5 and 10 wt% molasses solutions, respectively. At the start of the experiment the feed was heated under recirculation to 60 °C. The LCM experiments were performed at crossflow velocities (CFV) of 5, 4, and 1 m/s (turbulent flows) set points and in the transmembrane pressure (TMP) range of 2–12 bar. The trials started at 1 bar and CFV 5 m/s. The pressure was then increased, but the CFV was maintained constant. After all pressures at set CFV 5 m/s were tested, the CFV was reduced to 4 m/s at 1 bar pressure, and the pressure increase series for 4 and 1 m/s of CFV respectively was repeated. The retentate and permeate flow were both recirculated back to the feed tank. For each parameter set point, 20 mL of permeate was withdrawn for analysis. The samples were stored at −20 °C prior to analysis. The TMP was calculated as the average pressure between the feed and retentate side. 

The same procedure was repeated for the HCM experiments but with CFVs of 5, 3, and 1 m/s and a TMP range of 2–10 bar.

### 2.3. Experimental Setup of Ultrafiltration Trials

The UF parameter study was performed with a similar setup as the NF trial and approximates Figure 2 schematically, wherein the main difference from the NF experiments is that two centrifugal pumps were used in series instead of one displacement pump. A 50 L tank was used for the feed, equipped with a heating mantle. Two centrifugal pumps (FM-1A, Alfa Laval, Kolding, Denmark and CHI 2-60 A-W-G-BQQV, Grundfos, Bjerringbro, Denmark), connected in series, were used to create the desired flow, which was measured using a flowmeter (GPI S075, Great Plains Industries Inc., Wichita, KS, USA) on the retentate side of the membrane. Two frequency converters (SVS-252, Samco-V, Tyresö, Sweden and Commander CD 750, Control Techniques, Nidec Ltd., Shropshire, UK) was used to control the flow. The pressure was monitored by two pressure gauges (LE.C38-R2, BASI instruments AB, Vollsjö, Sweden and PA-35/10bar/80797, Keller AG, Winterthur, Switzerland) located at the feed and retentate sides of the membrane, respectively, and was controlled manually using a ball valve on the retentate side. The feed was preheated by connecting the feed tank mantle to a heating bath (601F, Julabo GmbH, Seelbach, Germany). The flux was determined by measuring the permeate flow on a scale (F150P-D2, Sartorius, Göttingen, Germany). 

Two concentrations of molasses were evaluated: HCM at 10 wt% and LCM at 1 wt% molasses. The molasses was diluted with deionized water to a total volume of 40 L, placed in the feed tank, and heated to 60 °C under recirculation. The experiment was conducted as in the NF test, by starting with a low TMP at a high CFV and increasing the TMP stepwise under constant CFV. The CFVs tested were 0.5, 1, and 1.5 m/s (turbulent flow region), in combination with a TMP range of 0.9–4.5 bar. The retentate and permeate flow were recirculated to the feed tank. 50 ml each of permeate and feed was sampled for each set point for analysis purposes. The samples were stored at −20 °C before analysis.

### 2.4. Fouling and Cleaning Studies

These studies were performed in different ways (amount of cleaning cycles, type of chemical, etc.), and the methods were thus divided by NF and UF test. The membranes were cleaned in different ways due to the aim to recover the capacity of the membrane to original levels. 

#### 2.4.1. Nanofiltration (NF) Fouling and Cleaning Sequence

As suggested by Trägardh, a 3-step cleaning sequence could improve the flux recovery and was thus used in this study [24]. Before the parameter study could be initiated, the new membrane was cleaned to wash out any impurities and preservation chemical present. First, 3 batches of 14 L deionized water was flushed through the system, after which an additional 14 L was added to the system with 1 wt% Ultrasil 110 (Ecolab AB, Älvsjö, Sweden), an alkaline cleaning chemical. The cleaning was then run at 50 °C at 3 bar pressure and 3 m/s of CFV for 1 h. Thereafter, the detergent solution was discarded, and the system was flushed with 3 batches of deionized water again, and pure water flux (PWF) was measured, based on the flux at 3, 5, 7, and 9 bar pressure at 30 °C and a CFV of 3 m/s. From these values, an average permeability was calculated (as flux per bar). 

Immediately after the parameter study with molasses, the system was emptied and flushed with 4 cycles of deionized water. Then, the change in membrane permeability was determined by measuring the PWF again, and the degree of fouling could be calculated, based on the % of permeability loss from the pristine membrane. The system was then cleaned using the same alkaline cleaning procedure as previously described. The PWF was measured for a third time, followed by an acid cleaning step using 0.5 wt% Ultrasil 73 (Ecolab AB, Älvsjö Sweden), at the same temperature, pressure, CFV, and time as for the alkaline cleaning step. The PWF was measured for a fourth time, followed by an alkaline cleaning step and a final PWF measurement. Before each PWF measurement, the system was flushed thoroughly with deionized water.

#### 2.4.2. Ultrafiltration (UF) Fouling and Cleaning Sequence

The membrane was initially cleaned with an enzymatic cleaning agent (Ultrasil 53, Ecolab, Älvsjö, Sweden) at a concentration of 1% in 30 L before any trials were conducted. The precleaning was performed at 40 °C, 1 bar pressure, and 0.5 m/s CFV under constant recirculation of the retentate and permeate for 1 h. The system was then emptied and flushed with deionized water. Thereafter, 25 L deionized water was added, and the PWF was determined at 1, 2, and 3 bar; 30 °C; and 0.5 m/s CFV. 

After the parameter study, the system was emptied and flushed thoroughly with deionized water. PWF was measured at the same set points as previously described. The membrane was then cleaned with the enzymatic cleaning agent under the same conditions as above, the system was washed thoroughly, and PWF was determined for a third time. This cleaning step was sufficient for the HCM test to recover good filtration capacity of the membrane. A second cleaning cycle was then performed after the LCM test, using the enzymatic cleaning agent again, and PWF was measured for a fourth time. In the final cleaning step, alkaline cleaning agent was used (Ultrasil 10, Ecolab, Älvsjö, Sweden) at 50 °C, 1 bar pressure, and a CFV of 0.5 m/s. A final measurement of PWF was made, and the permeability and degree of fouling could then be calculated. 

### 2.5. Analysis Methods

#### 2.5.1. Sugar Compounds

Sugar compounds were analyzed using a Dionex High-Performance Anionic-Exchange Chromatography (HPAEC) system ICS-5000+SP (Dionex Corp., Sunnyvale, CA, USA) that was equipped with a pulsed amperometric detection (PAD) detector, ICS-5000+DC pump, AS AP autosampler, and CarboPac PA20 carbohydrate analytical column. The eluent consisted of a flow mixture of 75% deionized water and 25% 200 mM NaOH at a flow rate of 0.5 mL/min and a flow rate of 0.25 mL/min with 200 mM NaOH as the post-column addition. The wash eluent was 200 mM NaOH with 170 mM sodium acetate, and the analysis temperature was 30 °C. The sample injection volume was 2.5 µL, and d-glucose, d-fructose, d-sucrose, and d-raffinose were used as calibration standards. 

#### 2.5.2. Total Solids and Ash

Total solids (TS) were measured using a Gallenkamp vacuum oven OVA03100 (Fistreem International Ltd., Leicestershire, UK). The samples were dried at 70 °C and 150 mbar pressure for 24 h, followed by 1 h cooling in a desiccator. The TS content was calculated as the difference in weight and was measured on a Precisa 410 AM-FR precision scale (Precisa Instruments Ltd, Dietikon, Switzerland). The residue was then heated in a muffle furnace (B150, Nabertherm GmbH, Lilienthal, Germany) at 700 °C for 3 h. Next, the samples were cooled in a desiccator for 1 h before being reweighed, and the ash content was determined. 

#### 2.5.3. Total Nitrogen and Nitrogen Salts

Total nitrogen (TN) measurements were performed on an N/Protein Analyzer (Flash EA 1112 Series, Thermo Electron S.p.A., Rodano, Italy), equipped with a carbon trap (soda lime), water trap (silica gel), catalysts of CuO and Pt/Al_2_O_3_, a separation column of Teflon and activated carbon, and a thermal conductivity detector (TCD). Dried samples were used per established TS protocols. In the analytical equipment, on dynamic flash combustion (Dumas method) of the samples, nitrogen compounds are released and reduced to nitrogen gas [25]. The analysis was performed at 900 °C, with helium as the carrier gas and oxygen gas was used for the combustion. Aspartic acid was used as the calibration standard.

The nitrogen salts that were analyzed included ammonia, nitrite, and nitrate. Spectrophotometry-based cuvette tests LCK 303, LCK 342 and LCK 340 (Hach Lange GmbH, Düsseldorf, Germany) were used to measure ammonia, nitrite, and nitrate, respectively. All cuvettes were analyzed on a DR2800 spectrophotometer (Hach Lange GmbH, Düsseldorf, Germany).

#### 2.5.4. Refractive Index, Turbidity, pH and Conductivity

Turbidity measurements was made using a 2100P ISO Turbidity meter (Hach Co., Loveland, CO, USA). Refractive index (RI) was measured on an HI96801 Refractometer (Hanna Instruments Inc., Woonsocket, RI, USA), in Brix. pH was measured using a HI8424 pH meter (Hanna Instruments Inc., Woonsocket, RI, USA), and conductivity was measured using a WTW Conductivity Meter LF95 (Christian Berner AB, Partille, Sweden).

#### 2.5.5. Starch

Starch was measured using the Megazyme Total Starch HK Assay Kit on dried molasses. The method used was the rapid total starch (RTS) method, a modified version by Megazyme of the standard AOAC method 996.11 [26,27]. This method does not account for resistant starch.

#### 2.5.6. Particle Size and Aggregates

For investigation of aggregate formation, changes in particle size distribution were studied by dynamic light scattering (DLS) using a Zetasizer Nano ZS (Malvern Instruments Ltd., Worcestershire, UK), with a scattering angle of 173°. The evaluation is based on averaged particle size measurements (Z-AVE) in different dilutions of crude molasses, ranging from 30% to 0.1% molasses in deionized water. This covers both the LCM and HCM concentrations and one data point above and below the selected region for more easily interpretation of trends in the physical phenomena. Four sets of data series were analyzed: filtered (0.2 µm syringe filters) and unfiltered molasses at 60 °C and 25 °C, respectively. Changes in Z-AVE, depending only on different dilutions of the molasses, were considered as potential aggregations.

#### 2.5.7. Organic Acids

Lactic acid and acetic acid were selected as potential inhibiting compounds and were, therefore, quantified in a Shimadzu high-performance liquid chromatography (HPLC) system, equipped with a Shimadzu RID 10A refractive index detector (Shimadzu Corporation, Kyoto, Japan). Samples were first pH adjusted to pH 4 using 10 wt.% H_2_SO_4_ and then filtered through a 0.2 um syringe filter. The organic acids were analyzed using a Bio-Rad Aminex HPX-87H column downstream a Cation-H Bio-Rad microguard column (Bio-Rad Laboratories, Hercules, CA, USA) by using a mobile phase of 5 mM H_2_SO_4_, at 50 °C and with a flow rate of 0.5 mL/min.

#### 2.5.8. Macromolecules

Macromolecules were analyzed using UV-absorption in a UV-1800 Shimadzu ultraviolet (UV) Spectrophotometer (Shimadzu Corporation, Kyoto, Japan). The measurements were performed at 280 nm wavelength, which has previously been used to measure for example proteins [28,29].

## 3. Results

### 3.1. Raw Material Composition

**Figure 3:**
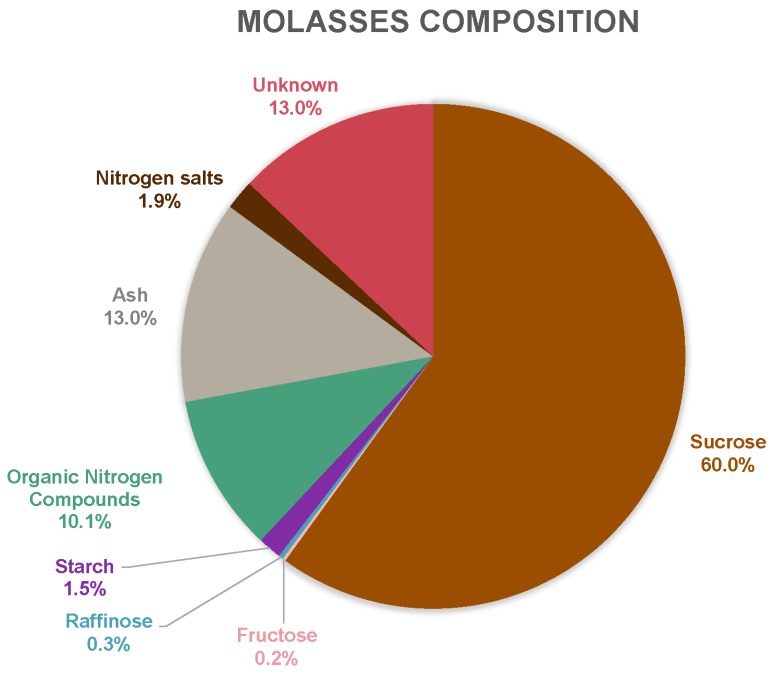
Composition of sugar beet molasses, based on total solids (TS) percentage.

The sugar beet molasses was provided by Örtofta sugar mill, located outside Lund, Sweden. The raw material composition of the molasses was analyzed (Figure 3) using the methods described in Section 2.5. The percentage is based on wt% of TS. Organic nitrogen compounds (assumed to be primarily proteins, amino acids, and betaine) was calculated by measuring TN, multiplying in by the Jones factor of 6.25 [30], and then subtracting the nitrogen salt value. Additional properties of interest are listed in Table 2. In comparison to previous work by Vučurović et al. and Steg et al., similar composition results were obtained [31,32]. Filipčev et al. suggested a few other compounds, which could explain some parts of the unknown category in Figure 3 [33]. 

### 3.2. Nanofiltration Parameter Study

**Figure 4:**
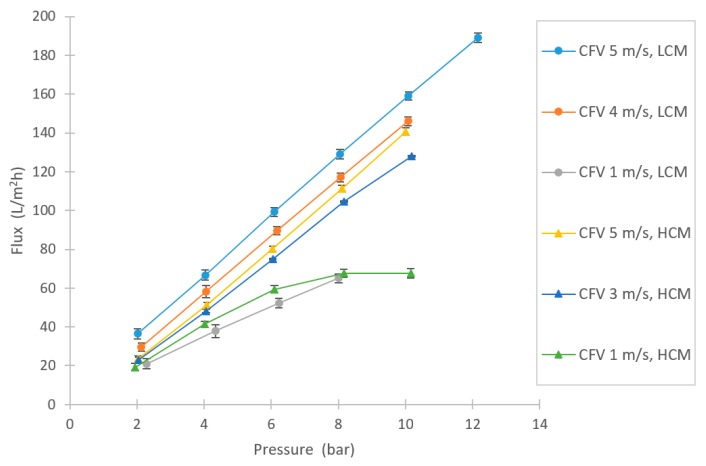
Varying flux in NF experiments at several different pressures and crossflow velocities (CFVs), for both the high-concentration molasses (HCM) and the low-concentration molasses (LCM).

One of the objectives in this study was to examine the filtration flux of the selected membrane for this specific application, based on pressure and CFV. As Figure 4 shows, high fluxes could be achieved. None of the CFV series lay in any limiting regions due to fouling, except for the HCM at 1 m/s, at which point the curve levels out at approximately 8 bar pressure [34,35]. Higher filtration fluxes were achieved with the LCM compared to the HCM, as expected. However, the difference in flux between the dilutions was not as remarkable as the difference in changing CFV, which implies that the concentration polarization resistance is an important factor for the filtration capacity. 

Since molasses consists of many different compounds including a relative large unknown fraction (see Figure 3), several analytical measurements were performed to obtain a broad overview of the retentions, defined and calculated according to Singh and Heldman [36]. However, for the NF experiments, only the low-molecular-weight compounds were of interest to measure due to the 200 Da MWCO of the membrane. Therefore, retentions in conductivity (as a measure of ions) and RI (as a measure of sugars and TS) were of interest to determine. 

**Figure 5:**
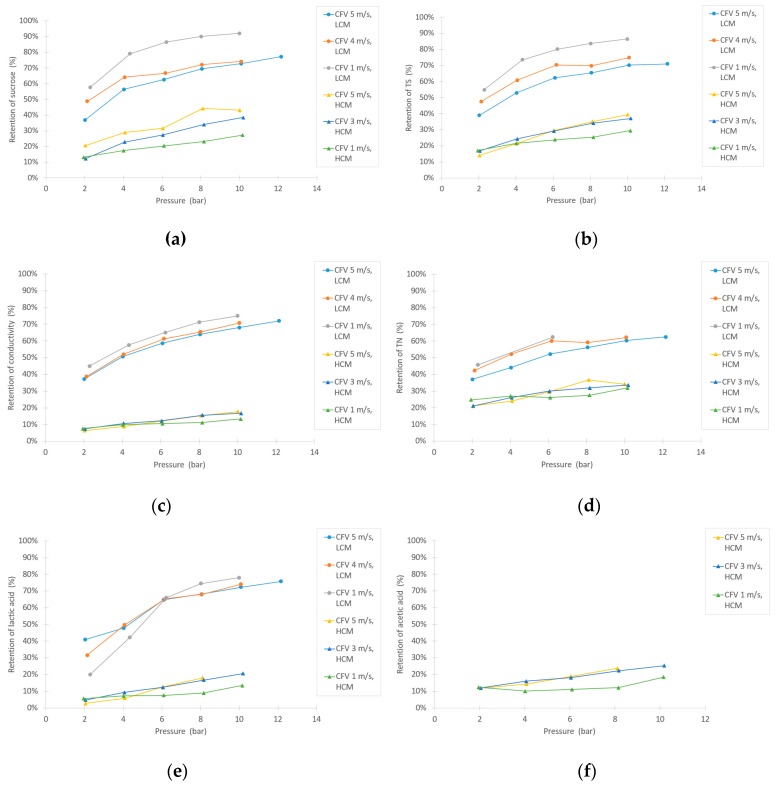
Retention of (**a**) sucrose, (**b**) TS, (**c**) conductivity, (**d**) total nitrogen (TN), (**e**) lactic acid, and (**f**) acetic acid during the NF experiments.

As shown in Figure 5a, the retention of sucrose was high for the LCM tests and remarkably lower for the HCM experiments. Overall, the retention increased with pressure, but when correlating the retention with CFV, two opposing trends were observed between the LCM and HCM tests. The TS retention increased with higher pressure, as shown in Figure 5b, and remained similar to the retention of sucrose, implying that the majority of the retained TS was sucrose. The turbidity and ˚Brix retentions were only measured for the HCM experiment, because the LCM was too dilute to obtain reliable results. As expected, the turbidity reached very high levels of retention (96% to 99%), whereas the °Brix retention rose slightly with increasing pressure, following a similar trend as the sucrose retention. The retention of conductivity (Figure 5c) had similar trends as the sucrose with respect to pressure and CFV. The retention of conductivity, however, were slightly lower than for sucrose, indicating that there is a small degree of purification of sucrose by the NF membrane and the many compounds that contribute to the conductivity are of low molecular sizes. In comparison with the findings of Linde and Jönsson, who purified leachate water using NF, they found similar patterns of decreased retention of conductivity when increasing the concentration of NaCl [37]. The TN retention (Figure 5d) also increased with pressure. This trend is very similar but slightly lower than the TS and sucrose retentions. It should be noted that there is also a remarkable difference between the TN retention of LCM compared to HCM. 

The compounds representing small inhibiting compounds are lactic acid and acetic acid. As shown in Figure 5e,f, the retention of these small compounds seems to follow the LCM vs. HCM trends as previously described. Acetic acid is only displayed for HCM, due to the lower detection limitations in the HPLC. In comparison to the sucrose retention, the retentions of lactic acid and acetic acid are not that different, indicating low purification of the sucrose. 

### 3.3. Ultrafiltration Parameter Study

**Figure 6:**
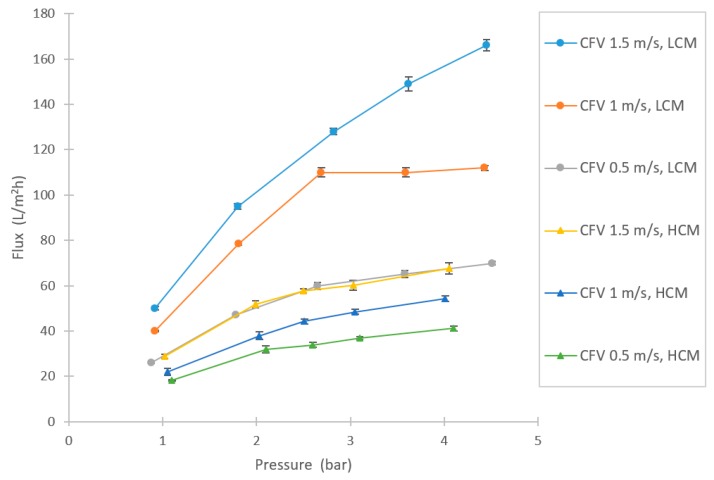
Flux variations at different setpoints of pressures and CFVs in the UF experiment for HCM and LCM.

In the UF tests, high filtration fluxes were achieved (see Figure 6). As expected, the LCM reached higher flux rates compared with HCM. The flux trends were generally not linear, implicating some limitation with the filtration capacity. Above 2.5 bar, the curves tended to level out somewhat (sustainable flux region, as described in Bacchin et al.), especially for LCM at CFV 1 m/s, which appeared to have reached the limited flux region [34,35]. 

**Figure 7:**
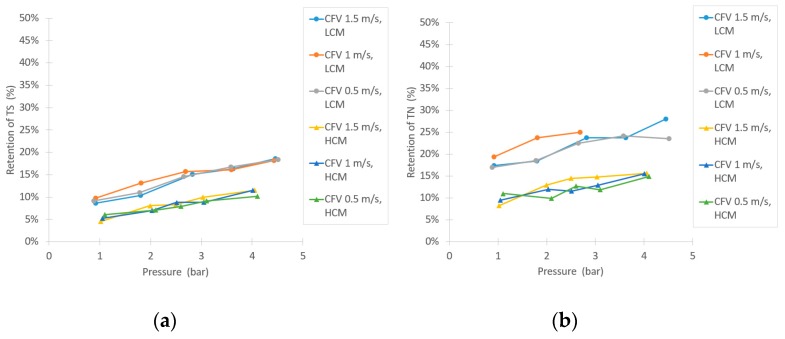

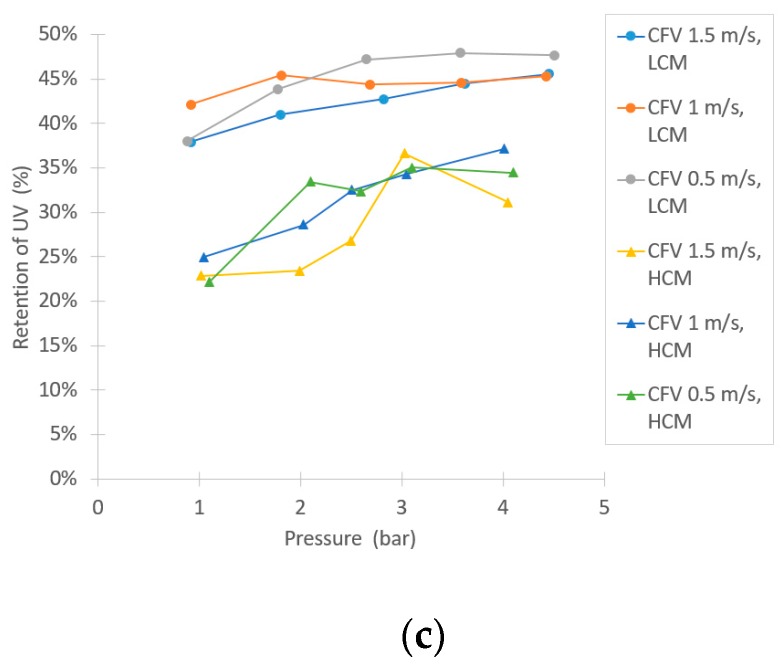
Separation efficiency results in the UF experiment, shown as: (**a**) TS retention, (**b**) retention of TN, and (**c**) retention in ultraviolet (UV) absorption.

Similar to the NF trials, there was a notable difference in retentions during UF of the LCM and HCM, as Figure 7 implies. The retention of sucrose was low, as expected, between 0–15% at the tested parameters for both the LCM and HCM and no major difference in sucrose retention between LCM and HCM was observed. The conductivity retention showed a small difference between LCM and HCM, but not as extensive as in the NF trials. There was also an increasing trend of retention following the increase of TMP. For the retentions of TS and TN, Figure 6b and Figure 7a, there are higher retentions during the filtration of the LCM compared to the HCM. These retentions also rose at higher TMPs. The compounds that were retained by UF are likely to be proteins, due to the relatively high TN content in the feed. As suggested by Ser et al., proteins can precipitate from a solution through dilution, which might explain the difference in retention between LCM and HCM [38]. Figure 7c shows the retention of large compounds, given by the UV-absorption measurement. The results show that there is a difference in retention between LCM and HCM, as well. However, from a sucrose purification point of view, the UF retains large compounds to a larger extent than the sucrose, as both the UV-absorption and TN results show (LCM more than HCM), providing a purified sucrose solution in the permeate flow.

### 3.4. Fouling and Cleaning

#### 3.4.1. NF Fouling and Cleaning

**Figure 8:**
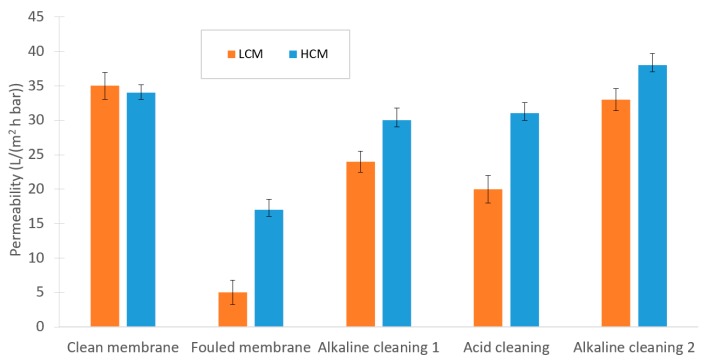
Changes in permeability during the experimental sequence of the NF trials.

As illustrated in Figure 8, fouling on the membrane differed considerably between the filtration of LCM compared to HCM. However, this alkaline-acid-alkaline cleaning sequence was necessary to recover the capacity of the NF membrane for both the LCM and the HCM. This result was in contrast to those of Jones et al., who used polymeric microfiltration membranes instead of ceramic NF for filtration of sugar beet molasses. However, they also experienced severe fouling on their microfiltration membranes [39].

#### 3.4.2. UF Fouling and Cleaning

**Figure 9:**
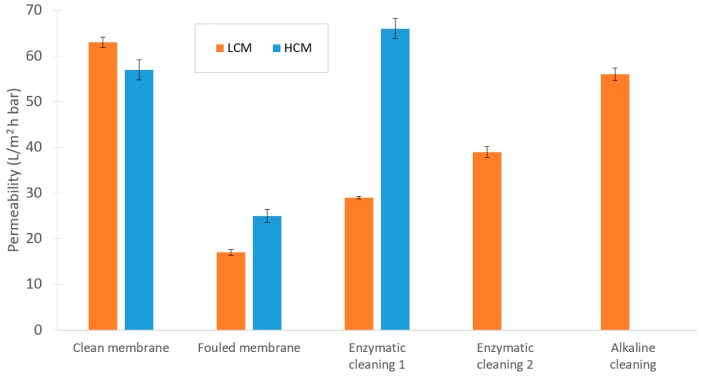
Changes in permeability during the experimental sequence in the UF trials.

Permeabilities were calculated based on the PWF measurements at 1, 2, and 3 bar. As the results in Figure 9 indicate, cleaning the membrane after the LCM test was more difficult compared with the HCM test. For the latter, one enzymatic cleaning cycle was sufficient, whereas the LCM required 3 cleaning steps to recover the filtration capacity of the membrane. Also, the fouling in the LCM test was slightly more severe. A similar study by Yang et al. showed that concentration polarization affects the total filtration resistance more than both the reversible fouling and the irreversible fouling when sugar cane molasses is filtered with a polymeric 10 kDa UF membrane. Although, they operated at different fluxes, concentrations, pressures, and shear forces than in this UF experiment, this could still provide possible indications of the impact of different kinds of fouling when filtering molasses in a UF [13].

### 3.5. Aggregate Formation

**Figure 10:**
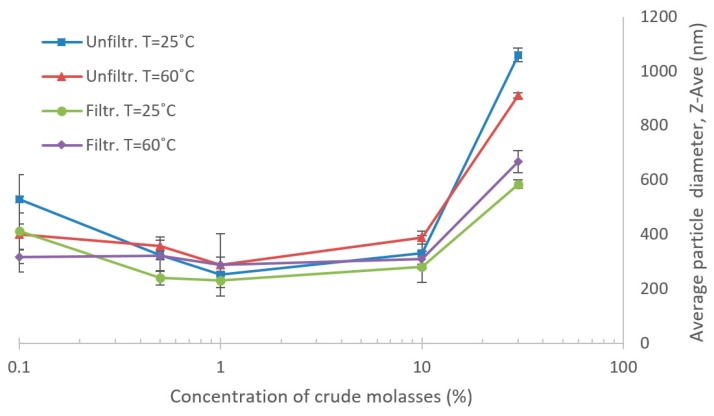
Average particle size of molasses at various concentrations. Samples that were passed through 0.2 µm filters and unfiltered samples were measured at 60 °C and 25 °C.

The retention results for the NF and UF trials suggest concentration-dependent properties of the solutes in the molasses, leading to unexpected differences in retention between the HCM and LCM tests. Thus, aggregate formation at various dilutions was investigated. The results of differently diluted crude molasses and the change in average particle diameter can be seen in Figure 10. The graphs indicate a tendency that the average particle size in molasses differ between dilutions. The average particle size seems to increase with rising concentration in the high-concentration range of the molasses. Also, in the dilute regions, the average particle size tends to increase at higher dilutions, implying some sort of aggregate formation. Furthermore, the impact of temperature was not so remarkable as the difference between filtered and unfiltered samples, especially in the HCM regions. 

## 4. Discussion

High flux rates were achieved in the NF and the UF trials, but the difference between filtering LCM and HCM in the UF test was more apparent. It was expected that the LCM would result in higher fluxes than HCM, due to its lower solids content. However, there was a remarkable difference in retentions between filtering LCM and HCM, especially during the NF experiment, in which the retention of sucrose is crucial to prevent product loss. The purification of sucrose from small inhibiting compounds (organic acids) by the NF membrane showed that, in relative values, the sucrose in the HCM is purified more than in the LCM, but the retention of sucrose in the HCM was not sufficiently high. The NF membrane is, therefore, not suitable for purifying the sucrose in the molasses for the downstream 5-HMF production process. The purification of sucrose from large molecular complexes by the UF membrane showed more promising results. Even though the LCM retentions were higher than for the HCM, with respect to TN and UV-absorption, these were suitable for the 5-HMF process.

Theoretically, higher concentration polarization for filtration of HCM versus LCM is expected when operating at similar mass transfer rates as in the NF trials [9,36,40]. Consequently, the difference between apparent and effective retention is larger due to the disparity between the concentrations in the bulk compared to the membrane surface, which differ for HCM and LCM with respect to the concentration gradients toward the membrane surface. This could also have affected the fouling, which, according to Figure 8 and Figure 9, was more severe for the filtration of LCM than for HCM. In comparison to a similar study on cane molasses by Gou et al., they explain some of the fouling by pore swelling of their polymeric membranes, which is not the case for the ceramic membranes used in this study [12]. Alternatively, Jones et al. suggest that the crystalline fouling on microfiltration membranes with sugar beet molasses comprises calcium sulfate and calcium oxalates [39]. It is also possible that for the HCM case, with higher ionic strength in the molasses, various salts could have flowed more easily through the membrane compared with LCM, due to a shielding effect [41]. However, the composition and type of fouling were not investigated further in this study.

The retention of sucrose, TS and TN appeared to follow each other, especially the sucrose and TS. If the purity is calculated as concentration sucrose per TS, the change in purity was not high. The purity of sucrose in the NF and UF trials was not as high as initially predicted, but for the UF test it was on a satisfactory level with respect to the TN results and the results obtained in the UV-absorption measurement. The TN retentions in the NF trials unexpectedly suggested that a significant part of the nitrogen compounds were of low molecular weight, such as amino acids or betaine [3]. The retention of small organic acids has a similar trend as the other compounds. Since these are small charged molecules, compared to sucrose which is uncharged, one would expect different retention behavior. However, the correlation between sucrose and TN retention implies that either they are in the same molecular sizes, or that some interactions occur between them, resulting in similar retention trends with respect to LCM and HCM tests. If there are interactions between sucrose and low-molecular nitrogen compounds, this could have an effect on both membrane fouling and aggregation phenomena. For the small organic acids and their interaction with sucrose, analogous reasoning as for the TN can be adopted.

As the results of the DLS suggest, there appears to be some form of aggregate formation at higher, and lower, dilutions of the molasses. It is not confirmed which compounds form these aggregates, whether there is only one phenomenon type of aggregation, or if it could possibly be caused by different types of physiochemical phenomena for the two regions of concentration, as seen in Figure 10 [42,43]. One theory, as previously mentioned, posits that the aggregates could be proteins that have precipitated during dilution [38]. However, the particle count rates were low in the DLS and ranged between 200–50 kcps, based on Z-AVE measurements of the crude molasses, which is a consequence of the high dilution factor. Furthermore, the molasses samples were also colored, which could cause some absorbance of the light and affect the results. Thus, the results obtained in the DLS should be considered as indicative and not as absolute values. Also, only aggregates that were rapidly formed were investigated. The effect of time was not considered in these DLS measurements, but could in the parameter studies perhaps be an influencing factor since the molasses had a retention time of several h at 60 °C [42]. Further research in this area is required to confirm aggregate formation, for instance a complementary particle size distribution analysis, and also what type of compounds that are aggregating.

Another possible explanation for the size differences in the DLS measurements and the difference in retentions, is molecular swelling due to changes in ionic strength. Similar to the proteins in a study by Cicuta and Hopkinson, it is possible that the size of the molecules in the molasses has changed during dilution, because the ionic strength is weakened [44]. 

## 5. Conclusions

It is possible to achieve high fluxes using ceramic NF and UF membranes when filtering sugar beet molasses. It is also possible to clean the membranes sufficiently after being heavily fouled to recover their filtration capacity. Increased fouling occurs when more dilute molasses is filtered, which also appears to affect the separation efficiency. The retentions were generally higher in the LCM versus HCM tests. This could be explained, in addition to fouling and concentration polarization, by protein derivate precipitation and aggregate formation, as the DLS results indicate. 

High sucrose retentions could be reached by the NF membrane, but the purification of the sucrose from various compounds was not sufficient. Therefore, this NF membrane is not suitable for our sucrose purification purposes. However, for the UF membrane, the sucrose purification from inhibiting compounds was considered as sufficient. Hence, the UF membrane will be considered as an alternative for the production of feedstock for future 5-HMF production trials. 

## Figures and Tables

**Table 1:** Overview of beet- and sugar cane molasses membrane studies.

Study by	Feed Type	Dilution Factor	Membrane Type and MWCO	Flux (L/m^2^/h)	Temp. (°C)	Pressure (bar)	Sucrose Retention (%)
Guo et al.	Cane molasses	3	Polymeric UF/NF, 0.4–5 kDa	17	60	5–10	10–90
Yang et al.	Cane molasses	6	Polymeric UF, 10–100 kDa	40	60	0.5–10	<10
Qiang et al.	Cane molasses	4–12	Ceramic UF/polymeric NF 300 kDa/400 Da	27–230	25–60	2–17	0–70
Luo et al.	Cane molasses	3+ (diafiltr.)	Polymeric NF (<97% MgSO_4_ rejection)	15	55–60	5–22	87–99
Goulas et al.	Model solution	-	Polymeric NF/RO, 1 kDa–96% MgSO_4_ rejection	10–110	25–60	7–28	45–99
Bernal et al.	Beet molasses	10	Ceramic UF, 100 kDa	18–25	25	1	-
Zhao et al.	Model solution	-	Polymeric NF 150–250 Da	5–120	30	12–14	82–99
Bandini & Morelli	Model solution	-	Polymeric NF/RO, 300 Da–98% MgSO_4_ rejection	0–410	30–50	0–27	78–99 (maltose retention)
Kuhn et al.	Model solution	-	Polymeric NF 150–1000 Da	-	25	25	50–93
Kaseno & Kokugan	Cane molasses	2.33	Ceramic MF, 0.05 µm	0.1–0.4	35	1–7	<5
Ryan & Johnson	Cane molasses	3	Polymeric UF, 300 kDa	-	-	2	-
Djordjevic et al.	Beet molasses	1.7–2.1	Ceramic MF, 0.2 µm	9–114	50	2	-
Jones et al.	Beet molasses	-	Polymeric MF, 0.5–1.5 µm	35–56	60	3	-

**Table 2:** Properties of sugar beet molasses.

pH	TS	Conductivity	Turbidity
8.9	81%	93 mS/cm	1630 NTU

## Appendix A

**Table A1:** List of abbreviations.

Abbreviation	Description	Abbreviation	Description
5-HMF	5-Hydroxymethylfurfural	PAD	Pulsed Amperometric Detection
CFV	Crossflow Velocity	PWF	Pure Water Flux
DLS	Dynamic Light Scattering	RI	Refractive Index
HCM	High Concentration Molasses	RTS	Rapid Total Starch
HPAEC	High-Performance Anion-Exchange Chromatography	TMP	Transmembrane Pressure
HPLC	High-Performance Liquid Chromatography	TN	Total Nitrogen
LCM	Low Concentration Molasses	TS	Total Solids
MWCO	Molecular Weight Cutoff	UF	Ultrafiltration
NF	Nanofiltration	Z-AVE	Average Particle Size
